# Effects of Dietary n–3 and n–6 Polyunsaturated Fatty Acids in Inflammation and Cancerogenesis

**DOI:** 10.3390/ijms22136965

**Published:** 2021-06-28

**Authors:** Kamila P. Liput, Adam Lepczyński, Magdalena Ogłuszka, Agata Nawrocka, Ewa Poławska, Agata Grzesiak, Brygida Ślaska, Chandra S. Pareek, Urszula Czarnik, Mariusz Pierzchała

**Affiliations:** 1 Department of Genomics and Biodiversity, Institute of Genetics and Animal Biotechnology of the Polish Academy of Sciences, ul. Postepu 36A, Jastrzebiec, 05-552 Magdalenka, Poland; k.liput@igbzpan.pl (K.P.L.); m.ogluszka@igbzpan.pl (M.O.); a.nawrocka@igbzpan.pl (A.N.); e.polawska@igbzpan.pl (E.P.); 2 Department of Molecular Biology, Institute of Genetics and Animal Biotechnology of the Polish Academy of Sciences, ul. Postepu 36A, Jastrzebiec, 05-552 Magdalenka, Poland; 3 Department of Physiology, Cytobiology and Proteomics, West Pomeranian University of Technology, ul. K. Janickiego 29, 71-270 Szczecin, Poland; adam.lepczynski@zut.edu.pl (A.L.); agata.grzesiak@zut.edu.pl (A.G.); 4 Department of Experimental Genomics, Institute of Genetics and Animal Biotechnology of the Polish Academy of Sciences, ul. Postepu 36A, Jastrzebiec, 05-552 Magdalenka, Poland; 5 Institute of Biological Bases of Animal Production, Faculty of Animal Sciences and Bioeconomy, University of Life Sciences in Lublin, Akademicka 13, 20-950 Lublin, Poland; brygida.slaska@up.lublin.pl; 6 Department of Basic and Preclinical Sciences, Institute of Veterinary Medicine, Faculty of Biological and Veterinary Sciences, Nicolaus Copernicus University, ul. J. Gagarina 7, 87-100 Toruń, Poland; pareekcs@umk.pl; 7 Division of Functional Genomics in Biological and Biomedical Research, Centre for Modern Interdisciplinary Technologies, Nicolaus Copernicus University, ul. Wilenska 4, 87-100 Torun, Poland; 8 Department of Pig Breeding, Faculty of Animal Bio-Engineering, University of Warmia and Mazury in Olsztyn, ul. M. Oczapowskiego 5, 10-719 Olsztyn, Poland; czar@uwm.edu.pl

**Keywords:** PUFA, omega-3 fatty acids, omega-6 fatty acids, oxylipins, inflammation, cancerogenesis

## Abstract

The dietary recommendation encourages reducing saturated fatty acids (SFA) in diet and replacing them with polyunsaturated fatty acids (PUFAs) n–3 (omega–3) and n–6 (omega–6) to decrease the risk of metabolic disturbances. Consequently, excessive n–6 PUFAs content and high n–6/n–3 ratio are found in Western-type diet. The importance of a dietary n–6/n–3 ratio to prevent chronic diseases is linked with anti-inflammatory functions of linolenic acid (ALA, 18:3n–3) and longer-chain n–3 PUFAs. Thus, this review provides an overview of the role of oxylipins derived from n–3 PUFAs and oxylipins formed from n–6 PUFAs on inflammation. Evidence of PUFAs’ role in carcinogenesis was also discussed. In vitro studies, animal cancer models and epidemiological studies demonstrate that these two PUFA groups have different effects on the cell growth, proliferation and progression of neoplastic lesions.

## 1. Introduction

An improper diet can lead to negative metabolic changes, including increased blood pressure, overweight, obesity, elevated glucose, and cholesterol levels and leads to the development of chronic diseases, including cardiovascular diseases, cancer, diabetes and chronic respiratory diseases [1,2]. Dietary recommendations assume limiting the amount of consumption of saturated fatty acids (SFAs) due to their effect on increasing low-density lipoprotein (LDL) cholesterol, which contributes to an increased risk of developing cardiovascular disease (CVD). SFAs are also associated with the negative effect on tissue sensitivity to insulin, inflammation and lipid metabolism [3]. It is recommended that saturated fat intake accounts for less than 10% of the energy consumed per day [4]. However, in many countries, actual SFAs consumption usually exceeds the recommended value, e.g., in Canada, it reaches 10.4%, in some European countries 15.5%, and 11.0% in the USA [5,6,7]. A positive effect on health is observed when dietary SFA is replaced with polyunsaturated fatty acids (PUFAs) [8]. Such change in the diet has the potential to reduce the risk of cardiovascular disease and type II diabetes [3]. However, research conclusions from epidemiological studies are not the same. Meta-analysis of randomized controlled trials revealed that replacing SFA with mostly n–6 PUFAs is unlikely to reduce coronary heart disease (CHD) events, CHD mortality or total mortality [9]. Thus, the quality of PUFAs plays an important role in health effects.

## 2. N–6 to n–3 PUFAs in Diet

One of the first insights into the quality of PUFAs’ intake is based on the concept that the human genetic profile nowadays is very similar to the genes of our ancestors during the Paleolithic period 40,000 years ago. Thus, the n–6/n–3 ratio in our present diet should reflect the composition of human ancestors’ diet, in which the ratio of n–6/n–3 PUFAs was about 1:1 [10,11]. However, PUFAs’ biosynthesis from linoleic acid (LA, 18:2n–6). and α-linolenic acid (ALA, 18:3n–3) is not uniform among populations and is the result of the adaptation to nutrient exposure during *Homo sapiens* development [12]. Numerous studies demonstrated common genetic and epigenetic variations in genes-encoded key fatty-acid conversion enzymes, e.g., *ELOVL5, ELOVL2, FADS1* and *FADS2*, which determine the levels of PUFAs in human tissues [13,14,15]. The ancestral genotypic of fatty acid desaturase genes *FAD*S*1* and *FADS2* compared with present-day humans revealed two different *FADS* haplotypes—A and D, which differ in their ability to synthesize long-chain PUFAs. The haplotype D is more efficient in producing long-chain PUFAs, such as docosahexaenoic acid (DHA, 22:6n–3) and arachidonic acid (AA, 20:4n–6) from their precursors—LA and ALA—and has appeared on the lineage leading to modern humans. Nowadays, the distribution of *FADS* haplotypes is different in continents. Haplotype A is dominant in the American population, whereas a high frequency of haplotype D is present in Africa. Haplotype D more efficiently synthesizes LC-PUFAs from their precursors, which was advantageous to humans with limited access to AA and DHA [16]. The results of Martinelli et al. (2008) reported that *FADS* alleles associated with an elevated AA/LA in red blood cell (RBC) membranes were related to a greater risk of coronary artery disease (CAD) [17].

The concept of a balanced n–6/n–3 diet is also based on the results of the *fat-1* mouse model, which is genetically modified mice with *fat-1* genes derived from the non-parasitic nematode *Caenorhabditis elegans* and encodes n–3 desaturase. As a result, *fat-1* mice are able to convert n–6 to n–3 PUFAs. *Fat-1* transgenic mice are characterized by an increased content of n–3 PUFAs in their tissue, while the content of *n*–6 PUFAs is decreased. Consequently, the n–6/n–3 PUFAs ratio in tissue is about 1:1 in comparison to 20–50:1 in wild mice. This happens without using n–3 PUFAs supplementation [18,19].

The balance between n–6 to n–3 PUFAs in diet existed during the long evolutionary history of our genus. However, rapid dietary changes over short periods of time as have occurred over the past 100–150 years is a totally new phenomenon in human evolution. The human diet has changed over the last century. Following dietary recommendations, e.g., the American Heart Association Central Committee Advisory Statement from 1961, which promote SFA replacement with unsaturated acids, the consumption of vegetable oils, especially soybean oil, was increased in exchange for animal fats [20]. For example, in the diet of the residents of the United States of America, there was an increase in carbohydrate intake and a more than 1000-fold increase in soybean oil consumption, a rich source of LA. Consequently, the amount of the consumed LA increased, raising the ratio of LA to ALA (LA/ALA) of 6.4:1 in 1909 to 10.0:1 in 1999 [21].

The eating style characteristic for the population of developed countries, characterized by a high ratio of consumed n–6 acids to n–3 (15:1-20:1), is defined as the Western diet, in which LA is the dominant PUFA [20,22]. 

The recommended intake values are as follows: 0.5% of energy for ALA and 4% of energy for LA. However, ALA’s positive effect is observed for values higher than the recommended (0.6%–1% of energy) [3,8]. 

Table 1 shows the ratio of PUFAs n–6 to n–3 and LA/ALA consumed by different nations. However, there are several disadvantages of using the n–6/n–3 ratio, including unspecified fatty acids, quantified and not a unified expression of fatty acids abundance—molar (mol%) or mass terms expression (weight%, wt%) [23]. Another confusion in this field is raised by the fact that a variety of levels of long- and short-chain fatty acids result in identical n–6/n–3 ratios [24]. Thus, it should be emphasized that using the ratio of PUFAs n–6 to n–3 instead of the individual role of n–3 or n–6 FAs could be misleading. In addition, the ratio of saturated FA/unsaturated FA (SFA to UFA ratio) in the diet is also important for human health and should be also taken into account [25]. 

The importance of identifying the most optimal ratio of n–6 to n–3 PUFAs for humans is a crucial aspect, especially for the parenteral nutrition (PN) procedure. PN is a life-saving method of intravenous intake of caloric requirements of macronutrients and electrolytes, trace elements and vitamins delivery bypassing the gastrointestinal tract in case the gut is not accessible or usable, e.g., after surgical intestine resection [37]. One of the first types of lipid emulsion (LE) was based on 100% soybean oil (Intralipid^®^, Fresenius Kabi, Germany) contained a high amount of n–6 PUFAs, especially LA. The second generation of LE contains soybean oil with saturated medium-chain triglycerides. The third generation LE consists of 20% soybean oil and 80% olive oil. The fourth generation of LE are enriched by fish oil [38,39]. Some research suggests that phytosterols, a component of soy lipid emulsions, are a major health-complicating factor. However, in LE’s development, the ratio of n–6 to n–3 PUFAs decreased from n–6 to n–3 in soybean oils LE about 7:1 to a ratio around 2:1– 4:1 in fish oil LE [40]. Intravenous lipid emulsion that contains fish oil-based emulsions has been associated with the prevention of cholestasis and reversal of cholestasis [41].

## 3. The Importance of n–6 and n–3 PUFA in Humans

The very first researchers that showed the importance of polyunsaturated fatty acids in the diet were George and Mildred Burr. They performed studies on rats fed a special fat-free diet and observed several deficits in animals, including skin problems and disorders leading to death. These animals’ health status was improved after dietary administration of PUFAs—linoleic acid and α-linolenic acid [42,43,44].

Among others, Hansolaf Bang and Jørn Dyerberg contributed to discovering the positive effect of polyunsaturated fatty acids on health. In the seventies of the last century, their papers about reduced cholesterol, triglycerides, and pre-β-lipoproteins in the plasma of Greenland residents comparing to Danes and people of Greenlandic origin living in Denmark were published [45,46]. The diet of indigenous Greenlanders turned out to be richer in unsaturated fatty acids, particularly eicosapentaenoic acid (EPA, 20:5n–3) and DHA, with a higher PUFAs/SFAs ratio of 0.84 than 0.24 in the Danes’ diet. This finding was linked with available data of ischemic heart disease in the Greenlanders population at that time [47]. 

**Figure 1:**
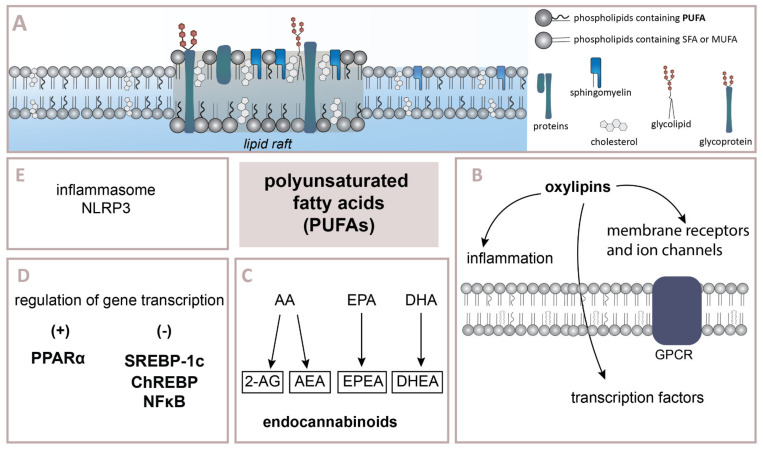
Roles of polyunsaturated fatty acids (PUFAs) in the cell biology: A) PUFAs are components of cell membranes and disk membranes of rod outer segment; B) PUFAs incorporated in phospholipids are sources of oxylipins—the lipid mediators, crucial in inflammation, gene transcription regulation and influence membrane G Protein-coupled receptors (GPCR) and ion channels; C) Endocannabinoids 2-arachidonoylglycerol (2-AG), anandamide (AEA), eicosapentaenoyl ethanolamide (EPEA) and docosahexanoyl ethanolamide (DHEA) derived from arachidonic acid (AA), eicosapentaenoic acid (EPA), docosahexaenoic acid (DHA), respectively, affect inflammation; D) PUFAs control gene transcription through regulation of transcription factors such as peroxisome proliferator-activated receptor α (PPARα) and sterol regulatory element binding protein-1c (SREBP-1c), carbohydrate-response element-binding protein (ChREBP), nuclear factor-κB (NFκB); E) N–3 PUFAs attenuate NLRP3 inflammasome activation. References: [60,61].

Polyunsaturated fatty acids play essential physiological functions in the body (Figure 1). NEFAs are an important source of energy production in the cell. NEFAs enter the mitochondrion in the reaction catalyzed by carnitine palmitoyltransferase 1 (CPT1). Then, they undergo β-oxidation, and in the form of acetyl-CoA becomes a substrate in the Krebs cycle. The newly emerging NADH/FADH2 are further converted to ATP by the mitochondrial electron transport chain [48,49]. Fatty acids are also incorporated into the cell membranes. Unsaturated fatty acids most often occur in the *sn*–2 position of cell membrane phospholipids. The incorporation of n–3 fatty acids, among others EPA and DHA, change the organization and size of lipid rafts [50,51]. EPA and DHA presence may also lead to increased membrane fluidity, but this has not been confirmed in the latest studies [52,53]. Additionally, fatty acids change cell membranes’ physiochemical properties by acting on membrane channels and G protein-coupled receptors (GPCR), which affects membrane permeability [54]. Five possible PUFA-binding sites in single ion channels have been demonstrated. PUFAs affect potassium, sodium and calcium channels’ functioning, especially in neurons and muscle cells [55,56]. 

Moreover, n–3 and n–6 PUFAs are precursors of endogenously produced cannabinoids (endocannabinoids, eCB), which are the ligands of the cannabinoid receptor 1 and 2 (CB1 and CB2). The CB1 receptors are localized mainly in the central nervous system, whereas the CB2 receptors are present, e.g., in immune cells such as B-lymphocytes and macrophages [57]. Anandamide (AEA) and 2-arachidonoylglycerol (2-AG) are eCB synthesized from AA. EPA and DHA (n–3 PUFAs) are precursors for eicosapentaenoyl ethanolamide (EPEA) and docosahexanoyl ethanolamide (DHEA), respectively. eCB can be metabolized by cyclooxygenases (COX), lipoxygenases (LOX) and cytochrome P450 enzymes (CYP). CYP converts AEA, EPEA and DHEA to EET-EA, EEQ-EA and EDP-EA, e.g., EET-EA, EEQ-EA and EDP-EA, which blocked pro-inflammatory interleukin IL-6 [58,59].

## 4. The Importance of n–3 and n–6 Fatty Acids in Regulating the Inflammatory Process

Inflammation is the body’s immune system response to tissue damage or infection. Depending on the duration, inflammation can be divided into acute (ending after a few or several days) and chronic. There are three main phases of acute inflammation: initiation, development and extinction of inflammation. The inflammatory reaction includes increased blood flow to the site of inflammation and an influx of leukocytes. Numerous mediators participate in inflammation, including cytokines, e.g., IL-1ra, IL-4, IL-6, IL-8, IL-10, IL-11, IL-13 and TGF-β [62,63].

The resolution of inflammation is an active process regulated by mediators and signaling pathways to prevent the development of chronic inflammation [64]. Polyunsaturated fatty acid oxidation products generated in non-enzymatic and enzymatic reactions have a potent influence on inflammation processes.

PUFAs are susceptible to free radicals because of the presence of double bonds. As a result, PUFAs are prone to free radical-induced autoxidation and photodegradation, leading to the generation of non-enzymatic metabolites including phytoprostanes (PhytoPs) generated from ALA, isoprostanes (IsoPs) generated from EPA and AA, dihomo-isoprostanes (dihomo-IsoPs) derived from AdA and neuroprostanes (NeuroPs) generated from DHA [65]. IsoPs, NeuroPs and PhytoPs are produced by uncontrolled oxidation are considered to be harmful biomarkers of oxidative damage in diseases [66,67]. IsoPs have been suggested as mediators of oxidative stress in the pathophysiology of chronic cardiovascular, respiratory, and metabolic diseases [68]. In spite of complex formation and metabolism, the F2-IsoPs are potential biomarkers because of their chemical stability in contrast to, e.g., MDA [69]. They also contain biological activities in humans and are competent to mimic the biological activity of enzymatic PUFA oxidation products; for instance, NeuroPs have potent anti-inflammatory activities similar to protectins [70]. Additionally, Campillo et al. provided evidence that individual PhytoPs have specific anti-inflammatory potential in vitro [71]. Moreover, PUFAs’ side chains of phospholipids are exposed to radical oxidation and generate oxidized phospholipid (OxPL) species, and one of them may contain IsoPs in the *sn-2* position. OxPL-derived isoprostanes have potent pro-resolving bioactivity [64].

**Figure 2:**
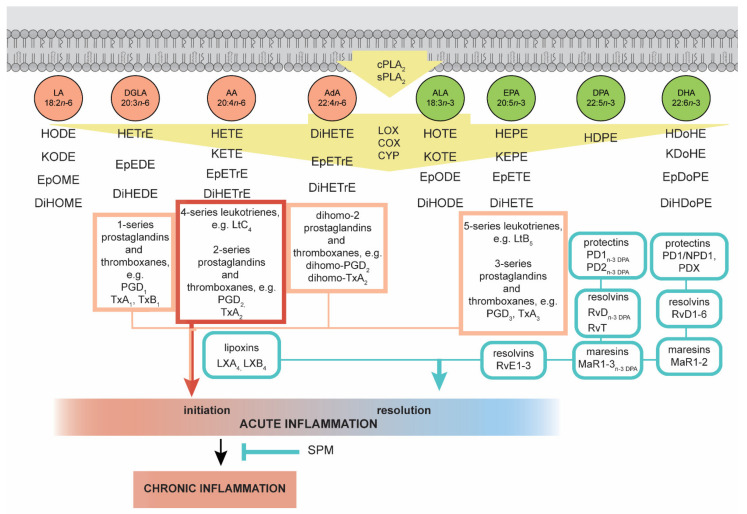
Lipid mediators enzymatically derived from n–3 and n–6 polyunsaturated fatty acids (PUFAs) and their role in inflammation. Acute inflammation response consists of three stages: initiation, development and resolution. In case of acute inflammation can lead to chronic inflammation. Polyunsaturated fatty acids released from phospholipids by cytosolic phospholipase A_2_ (cPLA_2_) and secreted phospholipase A_2_ (sPLA_2_) are converted by lipoxygenases (LOX), cyclooxygenases (COX) and cytochrome P450 (CYP) enzymes into bioactive oxylipins that act on inflammation. Specialized pro-resolving mediators (SPMs) can prevent the development of chronic inflammation. AA-derived oxylipins, such as prostaglandins, leukotrienes and thromboxanes, have pro-inflammatory proprieties. DGLA, AdA and EPA are precursors for less pro-inflammatory oxylipins. Major SPMs are synthesized from n–3 PUFAs. The blue marking indicates pro-resolving oxylipins. The red marking denotes pro-inflammatory lipid mediators. References: [67,72,73,79,80,81,82].

Enzymatically oxygenated PUFAs are broadly termed ‘oxylipins,’ which include a broad range of derivatives, including specialized pro-resolving mediators (SPMs) [67]. SPMs are a novel group of endogenously synthesized compounds from n–3 and n–6 groups of PUFAs. SPMs include lipoxins, resolvins, protectins and maresins [62,72]. PUFAs are oxidized by families of enzymes, including cyclooxygenases (COX), lipooxygenases (LOX) and cytochrome P450 (CYP) enzymes, resulting in the generation of inflammation-regulated oxylipins. In general, oxylipins generated from n–6 PUFAs have more potent pro-inflammatory and proliferative properties than oxylipins formed from n–3 PUFAs [73]. A dominant intake of n–6 PUFAs such as LA is linked to an increased concentration of LA-, DGLA-, AA-derived oxylipins. The same trends are present with a high n–3 PUFAs diet. Enhanced intake of ALA results in an increase in ALA-, EPA- and DHA-derived oxylipins [54,74]. A diet with a high LA/ALA ratio, e.g., 18.31:1 resulted in an increase in n–6/n–3 oxylipins in the liver in comparison to a lower LA/ALA ratio in a diet, e.g., 7.76:1 [75]. The production of oxylipins is initiated by the increase in intercellular calcium concentrations, which leads to translocation of cytosolic phospholipase A2 (cPLA_2_) to the cell membrane, where cPLA_2_ releases PUFAs from the sn-2 position of phospholipids [76]. Oxylipins can be classified according to their precursors: 1) octadecanoids formed from LA and ALA, 2) eicosanoids derived from AA, DGLA and EPA, 3) docosanoids derived from AdA, DPA and DHA [73,77,78] (Figure 2).

### 4.1. Oxylipins Derived from n–6 PUFAs

LA octadecanoids include hydroxy-octadecadienoic acids (HODEs) and dihydroxy-octadecenoic acid (DiHOMEs). 9-HODE and 13-HODE play a role in the pathogenesis of atherosclerosis and nonalcoholic steatohepatitis; however, other LA-oxylipins 13-oxo-ODE have anti-inflammatory properties [73,83]. DGLA is a precursor of 1-series prostaglandins and thromboxanes (PGI_1_, TxA_1_), as well as hydroxy-eicosatrienoic acids (HETrEs), e.g., 15-HETrE [73].

COX transforms AA to 2-series prostanoids, e.g., prostaglandin H_2_ (PGH_2_), which is a precursor of PGD_2_, PGE_2_, PGF_2α_ and PGI_2_ [60]. Moreover, COXs catalyze the transformation of AA into thromboxanes A_2_ (TxA_2_) and hydroxy-eicosatetraenoic acid (HETEs), e.g., 5-, 12-, 15-HETE, secreted by epithelial cells and leukocytes are linked with obesity, inflammation and cancer [73,82]. AA can also be oxygenated by CYPs, which generate 7-, 10-, 13- and 20-HETEs [84]. In addition, the epoxidation of AA by CYPs produces epoxy-eicosatrienoic acids (EpETrEs or EETs) and is further converted into dihydroxyeicosatrienoic acids (DiHETrE) [67].

It is worth mentioning that AA is also a precursor of anti-inflammatory and pro-resolving lipoxins, e.g., LXA_4_ i LXB_4_ [72]. Moreover, AA can be converted to 4-series of leukotrienes, e.g., LtC_4_ and hepoxilins (HEETAs or HXs) and then to trioxilins (THETAs or TrXs), which have neutrophils modulatory properties [85].

### 4.2. Oxylipins Derived from n–3 PUFAs

ALA-derived octadecanoids group include hydroxy-octadecatrienoic acids (HOTrEs) and dihydroxy-octadecatrienoic acids (DiHODEs), e.g., 13-HOTrE, 9,10-DiHODE, 9,16-DiHOTrE [73].

The EPA oxidized metabolites comprise 3-series prostaglandins (PGE3, PGD3, PGF3α and PGI3) and thromboxane A3 (TxA_3_), 5-series leukotrienes (LtA_5_, LtB_5_, LtC_5_, LtD_5_, LtE_5_) and E-series resolvins (RvE1, RvE2 and RvE3) [73,86,87].

Studies performed on mice and humans proved that during inflammation, leukocytes produce oxylipins derived from DPA (22:5n–3) such as D-series protectins (PD1_n–3 DPA_, PD2_n–3 DPA_), maresins (MaR1_n–3 DPA_, MaR2_n–3 DPA_) and resolvins of the D series (RvD1_n–3 DPA_, RvD2_n–3 DPA_, RvD5_n–3 DPA_) [80]. Moreover, DPA n–3 is also converted to 13-series resolvins (RvT) RvT1, RvT2, RvT3 and RvT4 by endothelial COX-2. These RvTs have anti-inflammatory, pro-resolving properties and stimulate phagocytosis during bacterial infections [81].

DHA (22:6n–3) is the precursor of resolvins of the D series (RvD1, RvD2, RvD3, RvD4, RvD5 and RvD6), which are synthesized by macrophages and neutrophils [88]. DHA-derived protectins, PD1/NPD1 and PDX, are formed in the action of 15-LOX. PD1/NPD1 is present in the blood, glial cells, neutrophils, T lymphocytes and retinal pigment epithelial cells [89,90]. SPMs from DHA are produced by macrophages—maresins such as MaR1 and MaR2 [91]. 

Oxylipins acts as ligands and directly interact with nuclear receptors including peroxisome proliferator-activated receptors — PPARα, PPARβ/δ and PPARγ, as well as interacts with G protein-coupled receptors (GPCRs) by indirect effects [92].

A new class of lipid mediators is named elovanoids (ELVs), e.g., ELV-N32 and ELV-N34, which are neuroprotective di-hydroxylated derivatives of very long-chain n–3 PUFAs. ELVs are synthesized in the brain and retinal pigment epithelial (RPE) cells by ELOVL4 [93,94,95]. 

Other novel families of SPM are produced during bacterial infection: 1) maresin conjugates in tissue regeneration (MCTR); 2) protectin conjugates in tissue regeneration (PCTR); 3) resolvin conjugates in tissue regeneration (RCTR) [96].

## 5. PUFAs and Their Roles in Carcinogenesis

Alteration in lipid metabolism is a characteristic feature of cancer stem cells (CSCs) [97]. However, the results of the PUFAs levels in cancer tissues are inconclusive. The results of Mika et al. suggest that PUFAs are preferentially incorporated and predominantly metabolized in the colorectal cancer cells, which contain more n–3 and n–6 PUFAs than normal intestinal mucosa. One of the possible causes is that PUFAs are essential for cell membrane phospholipids formation, which is necessary during the rapid proliferation of cancer cells [98]. In contrast, the results of Zhang et al. observed an increase in n–6 PUFAs level and a decrease in n–3 PUFAs content in phospholipids from CRC tissues in comparison to the adjacent normal tissue [99]. A decrease in n–6 PUFAs levels and an increase in n–3 PUFAs in colorectal cancer (CRC) tissue were already reported by Yang et al. [100].

On the other hand, the results of in vitro and animal studies give conclusions that PUFAs may have anti-cancer properties. The consumption of n–3 PUFAs gave some evidence about decreasing the risk of developing various cancers, including leukemia [101], breast cancer [102], colon cancer [103,104], prostate cancer [105] and melanoma [106]. Dietary n–3 PUFAs suppress the inflammatory process, stimulate apoptosis, inhibit metastasis and tumor proliferation, and upregulate antioxidant enzymes’ gene expression. In contrast, the consumption of n–6 PUFAs has a procarcinogenic effect correlated with increased ratios of eicosanoids [107]. The reason may be that n–3 and n–6 PUFAs have opposite effects on cancer development [108].

It should be also mentioned that n–6 PUFAs similar to n–3 PUFAs have also anticancer activity, e.g., LA suppresses cancer cell growth by inducing ROS production and mitochondrial damage [109].

Fatty Acid-Binding Protein (FABPs) facilitate FA trafficking, interacting with intracellular proteins, e.g., PPARs, as well as in regulating tissue lipid responses. As a result, FABSs participate in tissue homeostasis, as well as in disease pathogenesis [110]. Epidermal FABP (E-FABP) is upregulated by HFD and promotes inflammasome activation and cytokine production in macrophages in skin tissues [111]. E-FABP is also an anti-tumor factor that promotes interferon β (IFNβ) responses in tumor-associated macrophages [112]. E-FABP (FABP5) has a stronger affinity for n–3 PUFAs than the others [113]. Moreover, adipose/macrophage FABP (A-FABP, FABP4) mediates ROS-mediated pro-tumor macrophage death induced by n–3 PUFAs [114].

**Figure 3:**
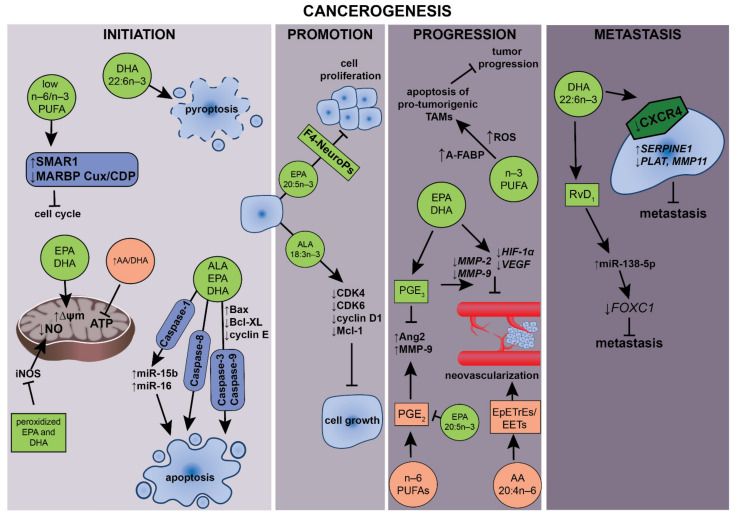
The effects of PUFAs related to the steps involved in the carcinogenic process. In the first stage of carcinogenesis, initiation: PUFAs regulate reactive oxygen species generation; EPA and DHA increase mitochondrial membrane potential (ΔΨm); Peroxidized EPA and DHA suppress iNOS followed by nitric oxide (NO) production; The increased AA to DHA ratio inhibits ATP production. In the second stage, n–3 PUFAs, including ALA, EPA and its derivatives such as F4-Neuroprostanes (F4-NeuroPs), are able to inhibit cancer cell growth and proliferation. During the progression stage: n–3 PUFAs and PGE_3_ influence tumor progression by inhibiting neovascularization by decreasing MMP, HIF-1α, VEGF and Ang2. Moreover, n–3 PUFAs are able to inhibit tumor progression through ROS-dependent apoptosis of pro-tumorigenic tumor-associated macrophages (TAMs). On the other hand, the n–6 PUFAs derivative, PGE_2_, increases Ang2 and metaloproteinaase-9 (MMP-9) expression, followed by inducing angiogenesis. Metastasis is also regulated by PUFAs by inducing apoptosis and pyroptosis by caspase pathway activation. The specialized pro-resolving mediator, RvD_1_, is able to inhibit metastasis.

The influence of PUFAs on carcinogenesis may occur through various mechanisms, which are presented in Figure 3.

### 5.1. Mitochondrial Activity

PUFAs alter cardiolipin’s fatty acid side chains and consequently cause mitochondrial phospholipid remodeling [115]. Supplementation with only DHA delayed Ca^2+^-induced mitochondrial permeability transition pore (MPTP) opening [115]. Treatments with the krill oil, which has a high concentration of phospholipids with EPA and DHA, resulted in a significant increase in the mitochondrial membrane potential [116].

A high ratio of n–6 to n–3 disrupts mitochondrial functions. According to Ghazali et al. (2020), a high AA/DHA ratio reduced mitochondrial activity, including decreased basal and maximal respiration, spare respiratory capacity, proton leak and ATP production [117]. 

Mitochondria are the potent source of cellular reactive nitrogen and oxygen species (RNS and ROS, respectively). mtNOS is considered the main source of RNS [118]. Mitochondrial complexes I (CI) and III (CIII) are the major sources of ROS within the mitochondria and the cell [119]. Mitochondria that produce ROS may generate oxidized phospholipids and isoprostanes, affecting inflammation and cancer progression.

Mitochondria is also a source of nitric oxide (NO). NO production is catalyzed by mitochondrial nitric oxide synthase (mtNOS) [120]. Nitric oxide (NO) and inducible NOS promote cancer development. Peroxidized products of EPA and DHA inhibited inducible NOS induction, followed by the reduction of NO production in proinflammatory cytokine-stimulated hepatocytes. The prevention of NO production is considered one of the indicators of anti-inflammatory effects [121].

### 5.2. Apoptosis and Cell Cycle

Apoptosis is a well-conserved mechanism of programmed cell death aimed to remove surplus or unnecessary, aged or damaged cells. Recently, PUFAs have been identified as important mediators for apoptosis modulation in brain tumors, e.g., EPA, together with radiation, can increase apoptosis in human C6 glioma cells [122]. DHA can strongly induce apoptosis in human MCF-7 breast cancer cells, which is selectively mediated via caspase 8 activation [123]. 

The activation of caspase-8-mediated apoptosis in ER+ MCF-7 cells was also confirmed after co-incubation with ALA. Moreover, ALA treatment arrested the cell cycle in the G2/M phase [124]. It was also confirmed that low ratios of n–6/n–3 (1:2.5 - 1:10) FA decreased the viability and growth of MDA-MB-231 and MCF7 breast cancer cell lines. Low n–6/n–3 PUFA ratios induced lipid peroxidation in the breast cancer cells, whereas the higher ratios of n–6/n–3 induced peroxidation in both cancerous and in non-tumorigenic human breast epithelial cells, which clearly shows the potential of n–3 PUFAs to semi-selective stimulation of apoptosis in cancerous cells. Interestingly, lower n–6/n–3 PUFAs ratios increased the expression of tumor suppressor SMAR1 and decreased the expression of tumor activator MARBP Cux/CDP in both breast cancer cell lines. Moreover, an increase in SMAR1 expression in cells treated with low n–6/n–3 PUFAs stimulates the expression of p21 protein that inhibits cell cycle progression [125].

The proapoptotic effect of EPA and DHA was also showed on MCF-7, SKBR-3 and MDA-MB-231 breast cancer cell lines. The authors observed an increased number of cells directed to cell arrest and a significantly increased apoptosis rate with simultaneous autophagy blockage after the treatment of the mentioned cell lines with the combination of EPA or DHA with Rapamycin. According to the authors, those effects were dependent on the increased ROS production from the intensified β-oxidation and oxidative phosphorylation in cancerous cells as a result of a metabolic switch caused by n–3 PUFA [126]. The mechanisms of ER-dependent DHA-mediated antiproliferative and proapoptotic in breast cancer cells were confirmed by Chénais et al. (2020). The results of their study confirmed that DHA mediates strong ER-stress response in MDA-MB-231, which was confirmed by the upregulation of numerous genes involved in heat shock and ROS stress and ER-stress induced apoptosis [127]. In another study, MDA-MB-231 breast cancer cells supplemented with DHA displayed an increased caspase-1 and gasdermin D activation, enhanced IL-1β secretion. Moreover, in these cells, a translocation of HMGB1 towards the cytoplasm and membrane pore formation were also showed what clearly indicates a pyroptosis-programmed cell death in breast cancer cells subjected to this n–3 PUFA [128].

The antiproliferative and proapoptotic effects of EPA and DHA were confirmed in vitro on colorectal cancer cell lines (human: DLD-1, HT-29, LIM-2405 and mouse CT-26). The treatment of cells with EPA, DHA and rich in those fatty acid krill oil caused an increased formation of ROS in all four cell lines as a result of changes of mitochondrial potential. Furthermore, the increase in ROS generation stimulated proapoptotic mechanism via changes of active forms of caspase-3 and caspase-9 expression [116]. Treatment of SGC7901 gastric cancer cells with DHA significantly induced apoptosis through suppressing Bcl-2 as well as activating caspase-9. Those effects were related to the increased proapoptotic miRNA: miR-15b and miR-16 expression. In vivo, DHA supplementation significantly inhibited the growth of SGC7901 cell-transplanted tumors [129].

The results of the study of Apte et al. (2013) aimed at the impact of different n–6 to n–3 ratios on prostate cancer progression clearly showed that a low ratio of those acids could lead to a delay in prostate cancer progression, including the development of castration-resistant tumor [130]. LNCaP PC cell lines under hormone deprivation were treated with medium with the addition of pure AA, pure DHA and the three ratios of AA:DHA (46:1, 10:1 and 1.3:1). The cells of the treatment independently respond to castration simulating hormonal deprivation and, subsequently, in the next 8-week period, showed an increase in proliferation in the case of the AA and AA:DHA ratios of 46:1 and 10:1. On the contrary, cells treated with a AA:DHA 1.3:1 ratio and pure DHA showed a constant proliferation decrease. Similar trends were observed for the activity of the PI3K/mTOR pathway, which was significantly higher after a 10-week treatment in a AA and AA:ADH 46:1 ratio and significantly decreased in the case of cells treated with AA:DHA 1.3:1 and DHA itself. The cells incubated with DHA and AA:DHA 1.3:1 tends to decrease in cyclin D1 expression. Oppositely, in the case of cells treated with DHA and AA:DHA 1.3:1, the highest expression of active caspase 3 was observed with a significant decrease in its expression in cells treated with AA that was reflecting apoptotic cell count. Similar to the previously described study [130], DHA induces apoptosis and apoptotic autophagy in in vitro cultivated prostate cancer PC-3 and DU145 cells in a mitochondrial ROS-regulated process that involves Akt-mTOR signaling [131].

A significant reduction of the metastatic potential of highly metastatic F10-SR cells B16 from melanoma F10 cell line was observed in mice fed a diet containing 5% of fish oil rich in EPA and DHA. The spreading potential was reduced not due to the diminished proliferation potential but as an effect of cell apoptosis [132].

Similarly, treatment of glioblastoma cell lines (D54MG, U87MG, U251MG and GL261) with DHA resulted in a significant increase in cells indicative of apoptosis and autophagic activity. This can be attributed to the effect of decreased levels of phosphorylated Akt and diminished mTOR activity. Moreover, in vivo studies on the *fat*-1 transgenic mice model, which have the ability to endogenous synthesize the n–3 fatty acids, yielded a significant decrease in tumor volume following implantation of mice glioma cells (GL261) when compared with wild-type mice [133].

DHA and EPA exhibited time- and concentration-dependent anti-proliferative effects on the other human brain cancer, neuroblastoma LA-N-1 cells. Those effects were related to G0/G1 cell cycle arrest accompanied by a decrease in the expression of cell cycle regulators: cyclin-dependent kinase 2 and cyclin E proteins. Moreover, both n–3 PUFAs were potent enough to induce apoptosis as a result of upregulation of Bax and downregulation of Bcl-XL proteins followed by subsequent activation of caspase-3 and caspase-9 [134].

DHA also potently inhibited growth in IGROV-1 ovarian cancer cells. Treatment with DHA resulted in G1 arrest and caused downregulation of cell cycle protein: CDK4, CDK6 and cyclin D1, and the antiapoptotic protein Mcl-1 [135].

The n–3 fatty acids are also able to reduce protein mass and induce pancreatic cancer cells apoptosis via the regulation of the WNT/β-catenin pathway. DHA and EPA significantly inhibited cell growth and increased cell death in pancreatic cancer cells (SW1990 and PANC-1) cultured in vitro. Moreover, coincubation with DHA also reduced β-catenin expression and induced β-catenin/Axin/GSK-3 complex formation, a known precursor of β-catenin degradation. In the in vitro study conducted on the tumor tissues from *fat*-1 transgenic mice inoculated with mouse pancreatic cancer cell line (PANC02), a significant increase in apoptosis rate was observed compared with those from inoculated control mice [84]. Similarly, the treatment of Hep3B, Huh-7 and HepG2 hepatocellular carcinoma cell lines with DHA and EPA resulted in inhibited cancerous cell growth through the simultaneous inhibition of COX-2 and β-catenin. Additionally, this treatment caused the activation of caspase-3 and caspase-9, which thereby mediated the apoptosis. DHA and EPA treatment caused dephosphorylation and thus activation of GSK-3β. Moreover, DHA induced the formation of β-catenin/Axin/GSK-3βbinding complex, which leads to β-catenin degradation. In contrast, AA exhibited none of the effects induced by *n*–3 PUFA [136]. 

Moreover, n–3 PUFA-enriched diets induce apoptosis in splenocytes, which is possibly mediated through the elevation of lipid peroxide levels and antioxidant enzymes [137].

### 5.3. Effects of n–3 PUFA on Tumor-Associated Macrophages and Cancer

PUFA and their roles in carcinogenesis are not limited to cancer cells but also influence the immune cells that are responsible for tumor development. The inflammatory process and cancer development are closely related. Chronic inflammation is a bipolar process that, on the one hand, may stimulate cancer development and progression, and, on the other hand, the recruitment of the immunocompetent cells and their activation may cause tumor suppression and apoptosis [138]. The important factor in tumor progression are tumor-associated macrophages (TAMs), especially alternatively activated subpopulations (M2) [139]. Tumor-associated cells are a potent source of immunomodulating molecules, i.a., pro-inflammatory cytokine IL-1, IL-6, TNF that are involved in the stimulation of key tumor-promoting factors as STAT3 and NF-κB [138,140]. Moreover, TAMs may cause changes in the tumor microenvironment by enzymes secretion, for example, MMPs, including MMP7, MMP2 and MMP9 [141]. It has been evidenced that ovarian cancer TAMs are characterized by significantly upregulated PPARβ/δ target genes in comparison to monocyte-derived macrophages. It was further confirmed using a lipidomic strategy that linoleic acid derivatives are potent PPARβ/δ agonists. The accumulation of those mediators in the intracellular vesicles of macrophages may be responsible for the pro-tumorigenic polarization of TAMs [142]. It may suggest that a high intake of LA that belongs to n–6 PUFA may be associated with ovarian cancer progression. On the other hand, the n–3 PUFA are exhibiting a significant ability to regulate TAMs population and activity. The development of mammary cancer (A0771 cells orthographically transplanted) in the mice model is increased by the dietary-induced obesity as an effect of the high cocoa butter consumption when compared to low-fat chow-fed animals. Moreover, the cancer development is assisted with the increased TAMs infiltration. Feeding the mice with an HFD based on the fish oil did not cause significant tumor growth. However, it has decreased the number of pro-tumorigenic macrophages. This phenomenon was an effect of the significant ROS generation and subsequent TAM’s apoptosis in the mechanism derived by adipose/macrophage-fatty acid-binding protein [114]. In the study of Liang et al., it was shown that in the case of the mice allografted with cancer androgen-sensitive prostate cancer cells fed with a high-fat diet (HFD) rich in n–3 FA TAMs proinflammatory cytokines (IL-6, TNF alpha and IL-10) and the chemoattractant protein (CCL-2) were lower expressed when compared to the animals fed an HFD based on n–6 FA [143]. The n–3 FA supplementation significantly reduced macrophage colony-stimulating factor receptor, responsible for transformation and recruitment of macrophages, in the TAM M2-like in the castration-resistant prostate cancer mice model. Moreover, n–3 FA reduced M2 macrophages towards MycCaP prostate cancer cells [144]. Additionally, in the in vitro experiment, it was evidenced that TAMs may stimulate the expression of MMPs (MMP1, 3 and 10) and migration of gastric cancer cells. Treatment with DHA and EPA significantly reduced macrophage-enhanced migration potential and MMP10 expression in the above-mentioned model [145]. 

### 5.4. Antiangiogenic and Antimetastatic Effects

Metastasis is the leading cause of death from most cancers. A major factor of metastasis is the migration of cancerous cells to other tissues by way of upregulated chemokine receptors, cell epithelial–mesenchymal transformation that causes a loss of cell polarity and cell–cell adhesion, and a gain of migratory and invasive phenotypes [146].

Additionally, neovascularization is one of the mechanisms responsible for neoplastic cell metastatic spreading [147]. One of the mechanisms involved in the regulation of tumor neoangiogenesis is hypoxia-induced up-expression of HIF-1α and the subsequent increase in VEGF synthesis [148]. The other significant factor necessary to promote neovascularization is related to metalloproteinases (MMPs) activity. MMPs belong to zinc-dependent endopeptidases, which specifically hydrolyze extracellular matrix (ECM) components and contribute to angiogenesis [149].

It has been demonstrated that n–6 PUFAs stimulate and n–3 PUFAs inhibit major proangiogenic processes in human endothelial cells, including the induction of angiopoietin-2 (Ang2) and metalloproteinase 9 (MMP-9), endothelial invasion and vessel formation. The COX-mediated conversion of PUFAs to prostanoid derivatives participate in the modulation of Ang2 expression. Thus, the n–6 PUFA-derived PGE_2_ augmented, whereas the n–3 PUFA-derived PGE_3_ suppressed the induction of Ang2 by growth factors [150]. 

Moreover, the antiangiogenic effect of n–3 PUFAs in different cancers has been proven up to date. The treatment of HT-29 colorectal cancer cell lines with EPA and DHA caused a decrease in VEGF expression via reduction of HIF-1α overexpression; however, more spectacular effects were observed after DHA treatment. In the in vivo model with orthographically transplanted HT-29 in mice, both n–3 PUFAs have reduced tumor size, micro-vessel formation and levels of VEGF [104].

On the other hand, suppressed expression of MMP-9 and MMP-2 and decreased lung metastasis rate in the individuals with colon cancer after supplementation with DHA have been shown by Suzuki et al. (1997) and Iigo et al. (1997) [151,152].

Moreover, a decreased expression of both MMP-1 and MMP-2 in gastric cancer tumors in patients treated with cisplatin and supplemented with PUFAs was also observed [153]. On the other hand, in the study aimed to highlight the effects of LA stimulation in OCUM-2MD3 gastric carcinoma cells in vitro, Matsuoka et al. (2010) found that metabolites of this n–6 FA caused an increase in phosphorylation of extracellular signal-regulated kinase (ERK) as an effect of increased COX-1 activity, which resulted in increased invasiveness of the cells [154]. Those effects were also confirmed in vivo using a model of experimentally induced metastasis after allographic transplantation of OCUM-2MD3 cells into gastric walls of immunocompromised mice that were fed diets containing linoleic acid (LA) at 2% (LLA), 8% (HLA) or 12% (VHLA) by weight. The dietary treatment resulted in an increased number of tumor metastatic nodules in the HLA and VHLA groups. The VHLA group also displayed increased numbers of tumor nodules and higher total volume relative to LLA. Both liver invasion (78%) and metastasis to the peritoneal cavity (67%) were more frequent in the VHLA group compared with the LLA group (22% and 11%, respectively; P<0.03). Thus, the authors concluded that high LA intake might regulate invasiveness and metastasis of gastric cancer via the regulation of the COX-1/pERK pathway [154].

Similar results were demonstrated in immunocompetent mice with allograph Myc-CaP androgen-dependent prostate cancer cells transplantation. Animals received the n–3 or n–6 rich diet 48h after the castration and were sacrificed after tumor regrowth signs that demonstrate castrate-resistant prostate cancer (CRCP). Animals fed the n–3 PUFAs rich diet showed significantly reduced growth MMP-9 and VEGF in the CRCP in comparison to the animals fed a diet rich in n–6 PUFAs. Additionally, n–3 PUFAs supplementation inhibit cancer progression inducing tumor-associated M2-like macrophages and their CSF-1R, MMP-9 and VEGF expression when compared to n–6 PUFA treatment, and therefore, increased their potential to stimulate angiogenesis. Moreover, the migration of M2 macrophages towards Myc-CaP cells was reversed by DHA [144]. On the other hand, Angelucci et al. (2008) observed that AA is a potent mitogenic factor for PCa cells through the production of both 5-lipoxygenase (5-LOX) and cyclooxygenase-2 (COX-2) metabolites that were responsible for the regulation of the Bcl-2/Bax ratio and apoptosis induction [155]. Moreover, COX-2 activity stimulates the release of TGF-α, TNF-α and IL-1β by AA in PCa cells. Those factors resulted in increased proliferation of bone marrow stromal cells. Moreover, it has also stimulated the proliferation of osteoblasts and the expression of receptor activators for nuclear factor κ B ligand. According to the authors, this cross-talk between prostate cancer cells and bone marrow stromal cells is a possible molecular mechanism by which dietary n–6 fatty acids accumulating in bone marrow may influence the formation of PCa-derived metastatic lesions. The mentioned mechanism was evaluated in the study of Brown et al. (2006) [156]. The results show that arachidonic acid at concentration 5mM is a potent stimulator of malignant epithelial cellular invasion, which is targeted on bone marrow stroma. Most probably, this invasiveness is an effect of stimulation by PGE2 and is inhibited by the n–3 PUFAs EPA and DHA at a ratio of 1:2 with AA.

It seems that n−3 PUFAs and their metabolites suppress the activity of angiogenic and inflammatory factors and ultimately inhibit an excess of vascularization. These findings stay in accordance with the study of Zhang et al. [157]. Authors showed that epoxydocosapentaenoic acids (EDPs), which are lipid mediators produced by cytochrome P450 epoxygenases from DHA, inhibit VEGF and fibroblast growth factor 2-induced angiogenesis in vivo and suppress endothelial cell migration and protease production in vitro via a VEGF receptor 2-dependent mechanism in mice inoculated with Met-1 syngeneic mammary tumor cells. Moreover, the inhibition of soluble epoxide hydrolase, the enzyme responsible for rapid EDPs inactivation, stabilized EDPs in circulation, causing ∼70% inhibition of primary tumor growth and metastasis. Adverse to the described effects of EDPs, metabolites of arachidonic acid (EETs) stimulated angiogenesis and tumor progression [157]. Recently, a decrease in tumor growth under n–3 diet has been observed as an effect of an increase in cellular levels of EPA and its oxidized derivates F4-neuroprostanes (F4-NeuroPs) and resolvins, suspected of anti-proliferative and anti-inflammatory properties, levels and reduced content of pro-angiogenic AA derivate PGE_2_ in castrated mice grafted with TRAMP-C2 prostate tumor cells [158].

As previously mentioned, metastasis is the leading cause of death from most cancers. The metastatic cell migration process requires, i.a., upregulated chemokine receptors, which serve as sensors for molecules enabling cell trafficking. It was previously shown that treatment of MDA-MB-231 breast cancer cells with DHA reduced surface expression of CXCR4, one of the chemokine receptors crucial for chemoattraction of metastatic cells, but also significantly reduced CXCR4-mediated cell migration. The above has led to the thesis that DHA may have a preventative effect on breast cancer metastasis in vitro. Novel mechanisms of DHA and its metabolite resolvin D1 (RvD1) on cancer cell growth and invasion were described by Bai et al. (2019) [159]. Moreover, DHA decreases MDA-MB-231 cells’ invasiveness via upregulation of *SERPINE1* and downregulation of *PLAT* and *MMP11* genes [127].

The authors demonstrated that the addition of exogenous DHA inhibited the growth and invasion in NSCLC (non-small cell lung carcinoma) cells in vitro. Moreover, a decrease in tumor growth and metastasis was also observed in vivo in transgenic *fat*-1 mice. Treatment with RvD1 significantly contributed to the inhibition of the cell growth and invasion via the increase in the miR-138-5p level, which significantly reduced the expression of *FOXC1*. *FOXC1* is responsible for the stimulation of mesenchymal transition and metastasis. In vivo endogenously synthetized DHA also enhanced the miR-138-5p expression and decreased the *FOXC1* expression. It also confirmed that dietary long-chain n–3 FAs modulates the mammary tumor microenvironment slowing tumor growth and reducing metastases to both preferred and less preferential organs, resulting in prolonged survival [160]. On the other hand, LA stimulation of the MDA-MB-231 breast cancer line induced an increase in fascin, which is an actin crosslinker globular protein that generates actin bundles and is involved in the stress fibers and filopodia formation. The invasive phenotype of MDA-MB-231 cells after LA stimulation exhibited the formation of filopodia and lamellipodia. Moreover, LA induced migration, invasion and matrix metalloproteinase-9 secretion through a fascin-dependent pathway [161].

Exposition of MDA-MB-231 and MCF-7 human breast cancer cell lines to fish oil high in DHA caused upregulation of PTEN tumor suppressor protein and subsequently inhibited the expression of CSF-1 (colony-stimulating factor-1) potent activator of malignancy and metastasis and its secretion from cancer cells through PI 3 kinase/Akt signaling. Moreover, DHA significantly inhibited the expression of miR-21, which is responsible for the stimulation of CSF-1 [162].

## 6. Conclusions

Increasing the PUFAs content only by increasing *n*–6 in the diet leads to a high ratio of n–6 to n–3 acids, which has a significant impact on the immune system. Evidence for the effect of PUFAs on the body’s immune response comes from numerous in vitro [163], animal [164,165] and human studies [166,167,168]. Actions and mechanisms of PUFAs on inflammation are multifactorial and are constantly investigated; among others, PUFAs acts through the oxylipins production and the regulation of transcription factors and epigenetic changes. The profiles of oxylipins in the body vary depending on the composition of fatty acids consumed. Multiomic studies on the transgenic mouse model have shown that the ratio of n–6/n–3 acids in tissues has a crucial impact on chronic diseases, including cancerous and inflammatory diseases, as well as affecting its microbiome. The therapeutic effect of a low ratio of n–6 to n–3 acids on the number of disorders has been demonstrated, e.g., colitis, melanoma, EtOH-mediated alterations in the gut-liver axis [169,170,171,172,173].

However, there are also opposite statements about the role of n–6 PUFAs on human health. According to Marangoni et al. (2020), LA intakes should be increased in most western countries [174]. An inverse association of both serum LA and AA with the risk of total and CVD mortality, as well as no association with cancer mortality, was found [175]. Additionally, the evidence based on a pooled global analysis of prospective observational studies demonstrated that higher in vivo circulating and tissue levels of LA were associated with a lower risk of total CVD, cardiovascular mortality, and ischemic stroke [176]. It was also demonstrated by Wu et al. that LA has long-term benefits for the prevention of type 2 diabetes and that arachidonic acid is not associated with the risk of the disease [177]. One of the possible reasons for numerous positive effects of LA on human health is the finding that dietary linoleic acid has no effect on arachidonic acid levels in plasma [178].

The proposed reason why results of scientific studies on the impact of consumption of PUFAs are not consistent is that number of previous cohort studies in the field of nutrition epidemiology took into account just the total content of PUFAs in the diet without differentiation into the group of n–3 and n–6 acids. Another reason for inconsistent results in some studies is the fact that the background diet and severity of disease were not taken into consideration. Moreover, there is a lack of reliable biomarkers associating PUFAs intake and risk of developing certain cancers because of the various methods of FA intake evaluation. A number of previous studies have used the FA levels in red blood cell (RBC) membranes to investigate the role of n–3 PUFAs on disease severity, whereas, according to the results of Moussa et al., PUFAs levels in RBC membranes are not associated with the grade prostate cancer. They showed that the EPA composition of prostate tissue is a reliable biomarker of prostate cancer risk [179]. Additionally, the mechanisms by which PUFAs remove cancer cells are multifaceted and include regulation of inflammation, reactive oxygen species level to induct the apoptosis cascade and mitochondrial function. Mechanisms of n–3 and n–6 PUFAs involved in tumor formation are mostly distinct. 

The key question is the optimal ratio of n–6 to n–3 acids, which will provide an effective immune response, and on the other hand, will it have a therapeutic effect in treating autoimmune diseases and chronic inflammatory diseases? It is also possible that the most favorable ratio of n–6 to n–3 acids in the diet may vary depending on the genetic background of PUFAs biosynthesis enzymes, e.g., FADS genes, age, gender and health condition. Another question is the potential benefits or risks of PUFAs supplementation in patients with cancer which is still studied. Personalized and population-based dietary recommendations of n–6 to n–3 PUFAs intake may be necessary to provide the healthiest approach to nutrition.

## Figures and Tables

**Table 1:** Daily intake of polyunsaturated fatty acids, α-linolenic acid (ALA) and linoleic acid (LA), calculated on their basis ALA/LA and n–6/n–3 in the diet of different age groups and nationalities.

Age Group	Fatty Acids Intake	USA		France		Japan		Poland	
			Reference		Reference		Reference		Reference
Children, young people	LA (g/day)	12.60	[26]	6.10–8.00	[27]	10.00	[28]	10.33	[29]
	ALA (g/day)	1.40		0.70–0.90		1.60		2.37	
	LA/ALA	9.00:1		8.71:1–8.89:1		6.25:1		4.36:1	
	n–6/n–3	9.20:1		n.a.		5.10:1		4.10:1	
adult	LA (g/day)	15.10 - 15.90	[30]	8.40	[31]	13.38–14.94	[32]	10.30	[33]
	ALA (g/day)	1.50 - 1.60		0.90		2.14-2.20		1.60	
	LA/ALA	9.94:1 - 10.07:1		9.33:1		6.25:1		6.44:1	
	n–6/n–3	n.a.		n.a.		3.70:1		5.78:1	
elderly	LA (g/day)	14.70	[26]	8.60–9.00	[34]	11.20–12.40	[35]	5.52 (men); 4.56 (women)	[36]
	ALA (g/day)	1.60		1.00		1.58–1.81		1.63 (men); 1.39 (women)	
	LA/ALA	9.19:1		8.60:1–9.00:1		6.84:1–7.10:1		3.39:1 (men); 3.28:1 (women)	
	n–6/n–3	7.80:1		n.a.		3.28:1–5.00:1		n.a.	

ALA—α-linolenic acid; LA—linoleic acid; LA/ALA—the ratio of LA/ALA intake per day; n–6/n–3—the ratio of intake n–6 to n–3 PUFAs; n.a.—data not available.

## Data Availability

Not applicable.

## References

[1] Nelson R.H. (2013). Hyperlipidemia as a Risk Factor for Cardiovascular Disease. Prim. Care Clin. Off. Pr..

[2] WHO (2018). Noncommunicable Diseases Country Profiles 2018.

[3] Lenighan Y.M., McNulty B.A., Roche H.M. (2019). Dietary fat composition: Replacement of saturated fatty acids with PUFA as a public health strategy, with an emphasis on α-linolenic acid. Proc. Nutr. Soc..

[4] Health.gov, U.S. Department of Health and Human Services, U.S. Department of Agriculture 2015–2020 Dietary Guidelines for Americans. https://health.gov/dietaryguidelines/2015/guidelines/.

[5] Eilander A., Harika R.K., Zock P. (2015). Intake and sources of dietary fatty acids in Europe: Are current population intakes of fats aligned with dietary recommendations?. Eur. J. Lipid Sci. Technol..

[6] Harrison S., Brassard D., Lemieux S., Lamarche B. (2019). Consumption and Sources of Saturated Fatty Acids According to the 2019 Canada Food Guide: Data from the 2015 Canadian Community Health Survey. Nutrients.

[7] Harika R.K., Eilander A., Alssema M., Osendarp S.J., Zock P. (2013). Intake of Fatty Acids in General Populations Worldwide Does Not Meet Dietary Recommendations to Prevent Coronary Heart Disease: A Systematic Review of Data from 40 Countries. Ann. Nutr. Metab..

[8] Szpondar L., Mojska H., Ołtarzewski M., Piotrowska K., Jarosz M. (2017). Normy żywienia dla populacji Polski. Normy Żywienia dla Populacji Polski.

[9] Hamley S. (2017). The effect of replacing saturated fat with mostly n-6 polyunsaturated fat on coronary heart disease: A meta-analysis of randomised controlled trials. Nutr. J..

[10] Simopoulos A.P. (2011). Evolutionary Aspects of Diet: The Omega-6/Omega-3 Ratio and the Brain. Mol. Neurobiol..

[11] Simopoulos A.P. (2006). Evolutionary aspects of diet, the omega-6/omega-3 ratio and genetic variation: Nutritional implications for chronic diseases. Biomed. Pharmacother..

[12] Chilton F.H., Dutta R., Reynolds L.M., Sergeant S., Mathias R.A., Seeds M.C. (2017). Precision Nutrition and Omega-3 Polyunsaturated Fatty Acids: A Case for Personalized Supplementation Approaches for the Prevention and Management of Human Diseases. Nutrients.

[13] Morales E., Bustamante M., Gonzalez J.R., Guxens M., Torrent M., Mendez M., Garcia-Esteban R., Julvez J., Forns J., Vrijheid M. (2011). Genetic Variants of the FADS Gene Cluster and ELOVL Gene Family, Colostrums LC-PUFA Levels, Breastfeeding, and Child Cognition. PLoS ONE.

[14] Xie L., Innis S.M. (2008). Genetic Variants of the FADS1 FADS2 Gene Cluster Are Associated with Altered (n-6) and (n-3) Essential Fatty Acids in Plasma and Erythrocyte Phospholipids in Women during Pregnancy and in Breast Milk during Lactation. J. Nutr..

[15] Koletzko B., Lattka E., Zeilinger S., Illig T., Steer C. (2010). Genetic variants of the fatty acid desaturase gene cluster predict amounts of red blood cell docosahexaenoic and other polyunsaturated fatty acids in pregnant women: Findings from the Avon Longitudinal Study of Parents and Children. Am. J. Clin. Nutr..

[16] Ameur A., Enroth S., Johansson Å., Zaboli G., Igl W., Johansson A.C., Rivas M.A., Daly M.J., Schmitz G., Hicks A. (2012). Genetic Adaptation of Fatty-Acid Metabolism: A Human-Specific Haplotype Increasing the Biosynthesis of Long-Chain Omega-3 and Omega-6 Fatty Acids. Am. J. Hum. Genet..

[17] Martinelli N., Girelli D., Malerba G., Guarini P., Illig T., Trabetti E., Sandri M., Friso S., Pizzolo F., Schaeffer L. (2008). FADS genotypes and desaturase activity estimated by the ratio of arachidonic acid to linoleic acid are associated with inflammation and coronary artery disease. Am. J. Clin. Nutr..

[18] Kang J.X. (2007). Fat-1 transgenic mice: A new model for omega-3 research. Prostaglandins, Leukot. Essent. Fat. Acids.

[19] Kang J.X., Wang J., Wu L., Kang Z.B. (2004). Transgenic mice: Fat-1 mice convert n-6 to n-3 fatty acids. Nature.

[20] Chilton F.H., Murphy R.C., Wilson B.A., Sergeant S., Ainsworth H., Seeds M.C., Mathias R.A. (2014). Diet-Gene Interactions and PUFA Metabolism: A Potential Contributor to Health Disparities and Human Diseases. Nutrients.

[21] Blasbalg T.L., Hibbeln J., Ramsden C.E., Majchrzak S.F., Rawlings R.R. (2011). Changes in consumption of omega-3 and omega-6 fatty acids in the United States during the 20th century. Am. J. Clin. Nutr..

[22] Husted K.S., Bouzinova E.V. (2016). The importance of n-6/n-3 fatty acids ratio in the major depressive disorder. Medicina.

[23] Harris W.S. (2018). The Omega-6: Omega-3 ratio: A critical appraisal and possible successor. Prostaglandins Leukot. Essent. Fat. Acids.

[24] Harris W.S. (2006). The omega-6/omega-3 ratio and cardiovascular disease risk: Uses and abuses. Curr. Atheroscler. Rep..

[25] Chen J., Li J., Liu X., He Y. (2021). Effects of dietary fat saturation level on growth performance, carcass traits, blood lipid parameters, tissue fatty acid composition and meat quality of finishing pigs. Anim. Biosci..

[26] Sheppard K.W., Cheatham C.L. (2018). Omega-6/omega-3 fatty acid intake of children and older adults in the U.S.: Dietary intake in comparison to current dietary recommendations and the Healthy Eating Index. Lipids Health Dis..

[27] Guesnet P., Tressou J., Buaud B., Simon N., Pasteau S. (2019). Inadequate daily intakes of n-3 polyunsaturated fatty acids (PUFA) in the general French population of children (3–10 years) and adolescents (11–17 years): The INCA2 survey. Eur. J. Nutr..

[28] Miyake Y., Tanaka K., Sasaki S., Arakawa M. (2011). Polyunsaturated fatty acid intake and prevalence of eczema and rhinoconjunctivitis in Japanese children: The Ryukyus Child Health Study. BMC Public Health.

[29] Stachura A., Pisulewski P.M., Kopeć A., Leszczyńska T., Bieżanowska-Kopeć R. (2009). Oszacowanie spożycia tłuszczów ogółem oraz kwasów tłuszczowych przez młodzież wiejską Beskidu Żywieckiego Żywność. Nauka. Technologia Jakość.

[30] Raatz S.K., Conrad Z., Johnson L.K., Picklo M.J., Jahns L. (2017). Relationship of the Reported Intakes of Fat and Fatty Acids to Body Weight in US Adults. Nutrients.

[31] Tressou J., Moulin P., Vergès B., Le Guillou C., Simon N., Pasteau S. (2016). Fatty acid dietary intake in the general French population: Are the French Agency for Food, Environmental and Occupational Health & Safety (ANSES) national recommendations met?. Br. J. Nutr..

[32] Shijo Y., Maruyama C., Nakamura E., Nakano R., Shima M., Mae A., Okabe Y., Park S., Kameyama N., Hirai S. (2019). Japan Diet Intake Changes Serum Phospholipid Fatty Acid Compositions in Middle-Aged Men: A Pilot Study. J. Atheroscler. Thromb..

[33] Harton A., Choroszewska A., Gajewska D., Myszkowska-Ryciak J. (2013). Spożycie wielonienasyconych kwasów tłuszczowych przez kobiety ciężarne. Probl. Hig. Epidemiol..

[34] Buaud B., Tressou J., Guesnet P., Simon N., Pasteau S. (2018). Inadequacy of n-3 polyunsaturated fatty acid dietary intakes in the general French population of elderly (65 to 79 years old): The INCA 2 survey. J. Aging Res. Clin. Pract..

[35] Matsuoka Y.J., Sawada N., Mimura M., Shikimoto R., Nozaki S., Hamazaki K., Uchitomi Y., Tsugane S. (2017). Dietary fish, n-3 polyunsaturated fatty acid consumption, and depression risk in Japan: A population-based prospective cohort study. Transl. Psychiatry.

[36] Skop-Lewandowska A., Kolarzyk E., Zając J., Jaworska J., Załęska-Żyłka I. (2016). The Structure of Fats and Fatty Acid Consumption in Elderly People with Cardiovascular System Diseases. Adv. Clin. Exp. Med..

[37] Bielawska B., Allard J.P. (2017). Parenteral Nutrition and Intestinal Failure. Nutrients.

[38] Raman M., Almutairdi A., Mulesa L., Alberda C., Beattie C., Gramlich L. (2017). Parenteral Nutrition and Lipids. Nutrients.

[39] Singh B.K.S., Narayanan S.S., Khor B.H., Sahathevan S., Gafor A.H.A., Fiaccadori E., Sundram K., Karupaiah T. (2020). Composition and Functionality of Lipid Emulsions in Parenteral Nutrition: Examining Evidence in Clinical Applications. Front. Pharmacol..

[40] Klek S. (2016). Omega-3 Fatty Acids in Modern Parenteral Nutrition: A Review of the Current Evidence. J. Clin. Med..

[41] Vlaardingerbroek H., Singh R., Stoll B., Benight N., Chacko S., Kluijtmans L., Kulik W., Squires E.J., Olutoye O., Schady D. (2014). New generation lipid emulsions prevent PNALD in chronic parenterally fed preterm pigs. J. Lipid Res..

[42] Burr G.O., Burr M.M. (1929). A new deficiency disease produced by the rigid exclusion of fat from the diet. J. Biol. Chem..

[43] Burr G.O., Burr M.M. (1930). On the nature and role of the fatty acids essential in nutrition. J. Biol. Chem..

[44] Burr G.O., Burr M.M., Miller E.S. (1932). On the fatty acids essential in nutrition. J. Biol. Chem..

[45] Bang H.O., Dyerberg J. (1972). Plasma Lipids and Lipoproteins in Greenlandic West Coast Eskimos. Acta Med. Scand..

[46] Bang H.O., Dyerberg J., Nielsen A.B. (1971). Plasma lipid and lipoprotein pattern in Greenlandic West-coast Eskimos. Lancet.

[47] Bang H.O., Dyerberg J., Sinclair H.M. (1980). The composition of the Eskimo food in north western Greenland. Am. J. Clin. Nutr..

[48] Fillmore N., Mori J., Lopaschuk G.D. (2014). Mitochondrial fatty acid oxidation alterations in heart failure, ischaemic heart disease and diabetic cardiomyopathy. Br. J. Pharmacol..

[49] Ly L.D., Xu S., Choi S.-K., Ha C.-M., Thoudam T., Cha S.-K., Wiederkehr A., Wollheim C.B., Lee I.-K., Park K.-S. (2017). Oxidative stress and calcium dysregulation by palmitate in type 2 diabetes. Exp. Mol. Med..

[50] Shaikh S.R., Kinnun J.J., Leng X., Williams J.A., Wassall S.R. (2015). How polyunsaturated fatty acids modify molecular organization in membranes: Insight from NMR studies of model systems. Biochim. Biophys. Acta Biomembr..

[51] Hou T.Y., McMurray D.N., Chapkin R.S. (2016). Omega-3 fatty acids, lipid rafts, and T cell signaling. Eur. J. Pharmacol..

[52] De Santis A., Varela Y., Sot J., D’Errico G., Goñi F.M., Alonso A. (2018). Omega-3 polyunsaturated fatty acids do not fluidify bilayers in the liquid-crystalline state. Sci. Rep..

[53] Yang X., Sheng W., Sun G.Y., Lee J.C.-M. (2011). Effects of fatty acid unsaturation numbers on membrane fluidity and α-secretase-dependent amyloid precursor protein processing. Neurochem. Int..

[54] Hussey B., Lindley M.R., Mastana S.S. (2017). Omega 3 fatty acids, inflammation and DNA methylation: An overview. Crit. Rev. Food Sci. Nutr..

[55] Boland L.M., Drzewiecki M.M. (2008). Polyunsaturated Fatty Acid Modulation of Voltage-Gated Ion Channels. Cell Biophys..

[56] Elinder F., Liin S.I. (2017). Actions and Mechanisms of Polyunsaturated Fatty Acids on Voltage-Gated Ion Channels. Front. Physiol..

[57] Brown I., Cascio M.G., Rotondo D., Pertwee R.G., Heys S.D., Wahle K.W.J. (2013). Cannabinoids and omega-3/6 endocannabinoids as cell death and anticancer modulators. Prog. Lipid Res..

[58] Dyall S.C. (2017). Interplay Between n-3 and n-6 Long-Chain Polyunsaturated Fatty Acids and the Endocannabinoid System in Brain Protection and Repair. Lipids.

[59] McDougle D.R., Watson J.E., Abdeen A., Adili R., Caputo M.P., Krapf J.E., Johnson R.W., Kilian K.A., Holinstat M., Das A. (2017). Anti-inflammatory ω-3 endocannabinoid epoxides. Proc. Natl. Acad. Sci. USA.

[60] Shen L., Yang Y., Ou T., Key C.-C.C., Tong S.H., Sequeira R.C., Nelson J.M., Nie Y., Wang Z., Boudyguina E. (2017). Dietary PUFAs attenuate NLRP3 inflammasome activation via enhancing macrophage autophagy. J. Lipid Res..

[61] Tourdot B.E., Ahmed I., Holinstat M. (2014). The emerging role of oxylipins in thrombosis and diabetes. Front. Pharmacol..

[62] Nowak J.Z. (2010). Przeciwzapalne „prowygaszeniowe” pochodne wielonienasyconych kwasów tłuszczowych omega 3 i omega 6. Postepy Hig. Med. Dosw..

[63] Sultani M., Stringer A., Bowen J.M., Gibson R.J. (2012). Anti-Inflammatory Cytokines: Important Immunoregulatory Factors Contributing to Chemotherapy-Induced Gastrointestinal Mucositis. Chemother. Res. Pr..

[64] Friedli O., Freigang S. (2017). Cyclopentenone-containing oxidized phospholipids and their isoprostanes as pro-resolving mediators of inflammation. Biochim. Biophys. Acta Mol. Cell Biol. Lipids.

[65] Galano J.-M., Lee Y.Y., Oger C., Vigor C., Vercauteren J., Durand T., Giera M., Lee J.C.-Y. (2017). Isoprostanes, neuroprostanes and phytoprostanes: An overview of 25 years of research in chemistry and biology. Prog. Lipid Res..

[66] Ahmed O.S., Galano J.-M., Pavlickova T., Revol-Cavalier J., Vigor C., Lee J.C.-Y., Oger C., Durand T. (2020). Moving forward with isoprostanes, neuroprostanes and phytoprostanes: Where are we now?. Essays Biochem..

[67] Hajeyah A.A., Griffiths W.J., Wang Y., Finch A.J., O’Donnell V.B. (2020). The Biosynthesis of Enzymatically Oxidized Lipids. Front. Endocrinol..

[68] Medina S., Gil-Izquierdo Á., Durand T., Ferreres F., Domínguez-Perles R. (2018). Structural/Functional Matches and Divergences of Phytoprostanes and Phytofurans with Bioactive Human Oxylipins. Antioxidants.

[69] Milne G.L. (2017). Classifying oxidative stress by F2-Isoprostane levels in human disease: The re-imagining of a biomarker. Redox Biol..

[70] Bosviel R., Joumard-Cubizolles L., Chinetti-Gbaguidi G., Bayle D., Copin C., Hennuyer N., Duplan I., Staels B., Zanoni G., Porta A. (2017). DHA-derived oxylipins, neuroprostanes and protectins, differentially and dose-dependently modulate the inflammatory response in human macrophages: Putative mechanisms through PPAR activation. Free Radic. Biol. Med..

[71] Campillo M., Medina S., Fanti F., Gallego-Gómez J.I., Simonelli-Muñoz A., Bultel-Poncé V., Durand T., Galano J.M., Tomás-Barberán F.A., Gil-Izquierdo Á. (2021). Phytoprostanes and phytofurans modulate COX-2-linked inflammation markers in LPS-stimulated THP-1 monocytes by lipidomics workflow. Free Radic. Biol. Med..

[72] Serhan C.N., Petasis N.A. (2011). Resolvins and Protectins in Inflammation Resolution. Chem. Rev..

[73] Gabbs M., Leng S., Devassy J.G., Monirujjaman, Aukema H.M. (2015). Advances in Our Understanding of Oxylipins Derived from Dietary PUFAs. Adv. Nutr..

[74] Caligiuri S.P.B., Love K., Winter T., Gauthier J., Taylor C.G., Blydt-Hansen T., Zahradka P., Aukema H.M. (2013). Dietary Linoleic Acid and α-Linolenic Acid Differentially Affect Renal Oxylipins and Phospholipid Fatty Acids in Diet-Induced Obese Rats. J. Nutr..

[75] Leng S., Winter T., Aukema H.M. (2017). Dietary LA and sex effects on oxylipin profiles in rat kidney, liver, and serum differ from their effects on PUFAs. J. Lipid Res..

[76] Yeung J., Hawley M., Holinstat M. (2017). The expansive role of oxylipins on platelet biology. J. Mol. Med..

[77] Astarita G., Kendall A.C., A Dennis E., Nicolaou A. (2015). Targeted lipidomic strategies for oxygenated metabolites of polyunsaturated fatty acids. Biochim. Biophys. Acta Mol. Cell Biol. Lipids.

[78] Godessart N., Camacho M., López-Belmonte J., Anton R., García M., de Moragas J.M., Vila L. (1996). Prostaglandin H-synthase-2 is the main enzyme involved in the biosynthesis of octadecanoids from linoleic acid in human dermal fibroblasts stimulated with interleukin-1beta. J. Invest. Dermatol..

[79] Careaga M.M., Sprecher H. (1984). Synthesis of two hydroxy fatty acids from 7,10,13,16,19-docosapentaenoic acid by human platelets. J. Biol. Chem..

[80] Dalli J., Colas R.A., Serhan C.N. (2013). Novel n-3 Immunoresolvents: Structures and Actions. Sci. Rep..

[81] Dalli J., Chiang N., Serhan C.N. (2015). Elucidation of novel 13-series resolvins that increase with atorvastatin and clear infections. Nat. Med..

[82] Shearer G.C., Newman J. (2009). Impact of circulating esterified eicosanoids and other oxylipins on endothelial function. Curr. Atheroscler. Rep..

[83] Choque B., Catheline D., Rioux V., Legrand P. (2014). Linoleic acid: Between doubts and certainties. Biochimie.

[84] Song K.-S., Jing K., Kim J.-S., Yun E.-J., Shin S., Seo K.-S., Park J.-H., Heo J.Y., Kang J.X., Suh K.-S. (2011). Omega-3-Polyunsaturated Fatty Acids Suppress Pancreatic Cancer Cell Growth in vitro and in vivo via Downregulation of Wnt/Beta-Catenin Signaling. Pancreatology.

[85] An J.-U., Song Y.-S., Kim K.-R., Ko Y.-J., Yoon D.-Y., Oh D.-K. (2018). Biotransformation of polyunsaturated fatty acids to bioactive hepoxilins and trioxilins by microbial enzymes. Nat. Commun..

[86] Duvall M.G., Levy B.D. (2016). DHA- and EPA-derived resolvins, protectins, and maresins in airway inflammation. Eur. J. Pharmacol..

[87] Yang P., Jiang Y., Fischer S.M. (2014). Prostaglandin E3 metabolism and cancer. Cancer Lett..

[88] Serhan C.N., Levy B.D. (2018). Resolvins in inflammation: Emergence of the pro-resolving superfamily of mediators. J. Clin. Investig..

[89] Balas L., Guichardant M., Durand T., Lagarde M. (2014). Confusion between protectin D1 (PD1) and its isomer protectin DX (PDX). An overview on the dihydroxy-docosatrienes described to date. Biochimie.

[90] Chen P., Fenet B., Michaud S., Tomczyk N., Véricel E., Lagarde M., Guichardant M. (2009). Full characterization of PDX, a neuroprotectin/protectin D1 isomer, which inhibits blood platelet aggregation. FEBS Lett..

[91] Dalli J., Serhan C.N. (2019). Identification and structure elucidation of the pro-resolving mediators provides novel leads for resolution pharmacology. Br. J. Pharmacol..

[92] Barquissau V., Ghandour R.A., Ailhaud G., Klingenspor M., Langin D., Amri E.-Z., Pisani D.F. (2017). Control of adipogenesis by oxylipins, GPCRs and PPARs. Biochimie.

[93] Bhattacharjee S., Jun B., Belayev L., Heap J., Kautzmann M.-A., Obenaus A., Menghani H., Marcell S.J., Khoutorova L., Yang R. (2017). Elovanoids are a novel class of homeostatic lipid mediators that protect neural cell integrity upon injury. Sci. Adv..

[94] Bazan N.G. (2018). Docosanoids and elovanoids from omega-3 fatty acids are pro-homeostatic modulators of inflammatory responses, cell damage and neuroprotection. Mol. Asp. Med..

[95] Jun B., Mukherjee P.K., Asatryan A., Kautzmann M.-A., Heap J., Gordon W.C., Bhattacharjee S., Yang R., Petasis N.A., Bazan N.G. (2017). Elovanoids are novel cell-specific lipid mediators necessary for neuroprotective signaling for photoreceptor cell integrity. Sci. Rep..

[96] Serhan C.N., Chiang N., Dalli J. (2018). New pro-resolving n-3 mediators bridge resolution of infectious inflammation to tissue regeneration. Mol. Asp. Med..

[97] Li H., Feng Z., He M.-L. (2020). Lipid metabolism alteration contributes to and maintains the properties of cancer stem cells. Theranostics.

[98] Mika A., Kobiela J., Pakiet A., Czumaj A., Sokołowska E., Makarewicz W., Chmielewski M., Stepnowski P., Marino-Gammazza A., Sledzinski T. (2020). Preferential uptake of polyunsaturated fatty acids by colorectal cancer cells. Sci. Rep..

[99] Zhang J., Zhang L., Ye X., Chen L., Zhang L., Gao Y., Kang J.X., Cai C. (2013). Characteristics of fatty acid distribution is associated with colorectal cancer prognosis. Prostaglandins Leukot. Essent. Fat. Acids.

[100] Yang K., Li H., Dong J., Dong Y., Wang C.-Z. (2015). Expression profile of polyunsaturated fatty acids in colorectal cancer. World J. Gastroenterol..

[101] Arita K., Kobuchi H., Utsumi T., Takehara Y., Akiyama J., Horton A.A., Utsumi K. (2001). Mechanism of apoptosis in HL-60 cells induced by n-3 and n-6 polyunsaturated fatty acids. Biochem. Pharmacol..

[102] Chen J., Stavro P.M., Thompson L.U. (2002). Dietary Flaxseed Inhibits Human Breast Cancer Growth and Metastasis and Downregulates Expression of Insulin-Like Growth Factor and Epidermal Growth Factor Receptor. Nutr. Cancer.

[103] Chamberland J.P., Moon H.-S. (2014). Down-regulation of malignant potential by alpha linolenic acid in human and mouse colon cancer cells. Fam. Cancer.

[104] Calviello G., Di Nicuolo F., Gragnoli S., Piccioni E., Serini S., Maggiano N., Tringali G., Navarra P., Ranelletti F.O., Palozza P. (2004). n-3 PUFAs reduce VEGF expression in human colon cancer cells modulating the COX-2/PGE 2 induced ERK-1 and -2 and HIF-1α induction pathway. Carcinogenesis.

[105] Lloyd J.C., Masko E.M., Wu C., Keenan M.M., Pilla D.M., Aronson W.J., Chi J.-T., Freedland S.J. (2013). Fish oil slows prostate cancer xenograft growth relative to other dietary fats and is associated with decreased mitochondrial and insulin pathway gene expression. Prostate Cancer Prostatic Dis..

[106] Yamada H., Hakozaki M., Uemura A., Yamashita T. (2019). Effect of fatty acids on melanogenesis and tumor cell growth in melanoma cells. J. Lipid Res..

[107] Zanoaga O., Jurj A., Raduly L., Cojocneanu-Petric R., Fuentes-Mattei E., Wu O., Braicu C., Gherman C.D., Berindan-Neagoe I. (2018). Implications of dietary ω-3 and ω-6 polyunsaturated fatty acids in breast cancer. Exp. Ther. Med..

[108] Huerta-Yépez S., Tirado-Rodriguez A.B., Hankinson O. (2016). Role of diets rich in omega-3 and omega-6 in the development of cancer. Bol. Med. Hosp. Infant Mex..

[109] Lu X., Yu H., Ma Q., Shen S., Das U.N. (2010). Linoleic acid suppresses colorectal cancer cell growth by inducing oxidant stress and mitochondrial dysfunction. Lipids Health Dis..

[110] Li B., Hao J., Zeng J., Sauter E.R. (2020). SnapShot: FABP Functions. Cell.

[111] Zhang Y., Li Q., Rao E., Sun Y., Grossmann M.E., Morris R.J., Cleary M.P., Li B. (2015). Epidermal Fatty Acid Binding Protein Promotes Skin Inflammation Induced by High-Fat Diet. Immunity.

[112] Zhang Y., Sun Y., Rao E., Yan F., Li Q., Zhang Y., Silverstein K.A.T., Liu S., Sauter E., Cleary M.P. (2014). Fatty Acid-Binding Protein E-FABP Restricts Tumor Growth by Promoting IFN-β Responses in Tumor-Associated Macrophages. Cancer Res..

[113] Bando Y., Yamamoto M., Sakiyama K., Inoue K., Takizawa S., Owada Y., Iseki S., Kondo H., Amano O. (2014). Expression of epidermal fatty acid binding protein (E-FABP) in septoclasts in the growth plate cartilage of mice. J. Mol. Histol..

[114] Liu L., Jin R., Hao J., Zeng J., Yin D., Yi Y., Zhu M., Mandal A., Hua Y., Ng C.K. (2020). Consumption of the Fish Oil High-Fat Diet Uncouples Obesity and Mammary Tumor Growth through Induction of Reactive Oxygen Species in Protumor Macrophages. Cancer Res..

[115] Stanley W.C., Khairallah R.J., Dabkowski E.R. (2012). Update on lipids and mitochondrial function: Impact of dietary n-3 polyunsaturated fatty acids. Curr. Opin. Clin. Nutr. Metab. Care.

[116] Jayathilake A.G., Kadife E., Luwor R.B., Nurgali K., Su X.Q. (2019). Krill oil extract suppresses the proliferation of colorectal cancer cells through activation of caspase 3/9. Nutr. Metab..

[117] Ghazali R., Mehta K.J., Bligh S.A., Tewfik I., Clemens D., Patel V.B. (2020). High omega arachidonic acid/docosahexaenoic acid ratio induces mitochondrial dysfunction and altered lipid metabolism in human hepatoma cells. World J. Hepatol..

[118] Akopova O., Kotsiuruba A., Кoркач Ю., Kolchinskaya L., Nosar V., Gavenauskas B., Serebrovska Z., Mankovska I., Sagach V. (2016). The Effect of NO Donor on Calcium Uptake and Reactive Nitrogen Species Production in Mitochondria. Cell. Physiol. Biochem..

[119] Hernansanz-Agustín P., Enríquez J. (2021). Generation of Reactive Oxygen Species by Mitochondria. Antioxidants.

[120] Litvinova L., Atochin D.N., Fattakhov N., Vasilenko M., Zatolokin P., Kirienkova E. (2015). Nitric oxide and mitochondria in metabolic syndrome. Front. Physiol..

[121] Araki Y., Matsumiya M., Matsuura T., Oishi M., Kaibori M., Okumura T., Nishizawa M., Takada H., Kwon A.-H. (2011). Peroxidation of n-3 Polyunsaturated Fatty Acids Inhibits the Induction of iNOS Gene Expression in Proinflammatory Cytokine-Stimulated Hepatocytes. J. Nutr. Metab..

[122] Montecillo-Aguado M., Tirado-Rodriguez B., Tong Z., Vega O.M., Morales-Martínez M., Abkenari S., Pedraza-Chaverri J., Huerta-Yepez S. (2020). Importance of the Role of ω-3 and ω-6 Polyunsaturated Fatty Acids in the Progression of Brain Cancer. Brain Sci..

[123] Kang K.S., Wang P., Yamabe N., Fukui M., Jay T., Zhu B.T. (2010). Docosahexaenoic Acid Induces Apoptosis in MCF-7 Cells In Vitro and In Vivo via Reactive Oxygen Species Formation and Caspase 8 Activation. PLoS ONE.

[124] Roy S., Rawat A.K., Sammi S.R., Devi U., Singh M., Gautam S., Yadav R.K., Rawat J.K., Singh L., Ansari M.N. (2017). Alpha-linolenic acid stabilizes HIF-1 α and downregulates FASN to promote mitochondrial apoptosis for mammary gland chemoprevention. Oncotarget.

[125] Mansara P.P., Deshpande R.A., Vaidya M.M., Kaul-Ghanekar R. (2015). Differential Ratios of Omega Fatty Acids (AA/EPA+DHA) Modulate Growth, Lipid Peroxidation and Expression of Tumor Regulatory MARBPs in Breast Cancer Cell Lines MCF7 and MDA-MB-231. PLoS ONE.

[126] Zhu S., Feng N., Lin G., Tong Y., Jiang X., Yang Q., Wang S., Chen W., He Z., Chen Y.Q. (2018). Metabolic Shift Induced by ω -3 PUFAs and Rapamycin Lead to Cancer Cell Death. Cell. Physiol. Biochem..

[127] Chénais B., Cornec M., Dumont S., Marchand J., Blanckaert V. (2020). Transcriptomic Response of Breast Cancer Cells MDA-MB-231 to Docosahexaenoic Acid: Downregulation of Lipid and Cholesterol Metabolism Genes and Upregulation of Genes of the Pro-Apoptotic ER-Stress Pathway. Int. J. Environ. Res. Public Health.

[128] Pizato N., Luzete B.C., Kiffer L.F.M.V., Corrêa L.H., de Oliveira Santos I., Assumpção J.A.F., Ito M.K., Magalhães K.G. (2018). Omega-3 docosahexaenoic acid induces pyroptosis cell death in triple-negative breast cancer cells. Sci. Rep..

[129] Sun H., Meng X., Han J., Zhang Z., Wang B., Bai X., Zhang X. (2013). Anti-cancer activity of DHA on gastric cancer—An In Vitro and In Vivo study. Tumor Biol..

[130] Apte S.A., Cavazos D.A., Whelan K.A., Degraffenried L.A. (2013). A Low Dietary Ratio of Omega-6 to Omega-3 Fatty Acids May Delay Progression of Prostate Cancer. Nutr. Cancer.

[131] Shin S., Jing K., Jeong S., Kim N., Song K.S., Heo J.Y., Park J.H., Seo K.S., Han J., Park J.I. (2013). The omega-3 polyunsaturated fatty acid DHA induces simultaneous apoptosis and autophagy via mitochondrial ROS-mediated Akt-mTOR signaling in prostate cancer cells expressing mutant p53. Biomed Res Int..

[132] Mannini A., Kerstin N., Calorini L., Mugnai G., Ruggieri S. (2008). An enhanced apoptosis and a reduced angiogenesis are associated with the inhibition of lung colonisation in animals fed an n-3 polyunsaturated fatty acid-rich diet injected with a highly metastatic murine melanoma line. Br. J. Nutr..

[133] Kim S., Jing K., Shin S., Jeong S., Han S.-H., Oh H., Yoo Y.-S., Han J., Jeon Y.J., Heo J.Y. (2017). ω3-polyunsaturated fatty acids induce cell death through apoptosis and autophagy in glioblastoma cells: In Vitro and In Vivo. Oncol. Rep..

[134] So W.W., Liu W.N., Leung K.N. (2015). Omega-3 Polyunsaturated Fatty Acids Trigger Cell Cycle Arrest and Induce Apoptosis in Human Neuroblastoma LA-N-1 Cells. Nutrients.

[135] West L., Pierce S., Yin Y., Fang Z., Zhou C., Bae-Jump V. (2018). Docosahexaenoic acid (DHA), an omega-3 fatty acid, inhibits ovarian cancer growth and adhesion. Gynecol. Oncol..

[136] Lim K., Han C., Dai Y., Shen M., Wu T. (2009). Omega-3 polyunsaturated fatty acids inhibit hepatocellular carcinoma cell growth through blocking beta-catenin and cyclooxygenase-2. Mol. Cancer Ther..

[137] Avula C.P.R., Fernandes G. (1999). Modulation of antioxidant enzymes and apoptosis in mice by dietary lipids and treadmill exercise. J. Clin. Immunol..

[138] Multhoff G., Molls M., Radons J. (2012). Chronic Inflammation in Cancer Development. Front. Immunol..

[139] Zhou J., Tang Z., Gao S., Li C., Feng Y., Zhou X. (2020). Tumor-Associated Macrophages: Recent Insights and Therapies. Front. Oncol..

[140] Hayashi T., Fujita K., Nojima S., Hayashi Y., Nakano K., Ishizuya Y., Wang C., Yamamoto Y., Kinouchi T., Matsuzaki K. (2018). High-Fat Diet-Induced Inflammation Accelerates Prostate Cancer Growth via IL6 Signaling. Clin. Cancer Res..

[141] Lin Y., Xu J., Lan H. (2019). Tumor-associated macrophages in tumor metastasis: Biological roles and clinical therapeutic applications. J. Hematol. Oncol..

[142] Schumann T., Adhikary T., Wortmann A., Finkernagel F., Lieber S., Schnitzer E., Legrand N., Schober Y., Nockher W.A., Toth P.M. (2015). Deregulation of PPARβ/δ target genes in tumor-associated macrophages by fatty acid ligands in the ovarian cancer microenvironment. Oncotarget.

[143] Liang P., Henning S.M., Schokrpur S., Wu L., Doan N., Said J., Grogan T., Elashoff D., Cohen P., Aronson W.J. (2016). Effect of Dietary Omega-3 Fatty Acids on Tumor-Associated Macrophages and Prostate Cancer Progression. Prostate.

[144] Liang P., Henning S.M., Guan J., Grogan T., Elashoff D., Cohen P., Aronson W.J. (2020). Effect of dietary omega-3 fatty acids on castrate-resistant prostate cancer and tumor-associated macrophages. Prostate Cancer Prostatic Dis..

[145] Wu M.-H., Tsai Y.-T., Hua K.-T., Chang K.-C., Kuo M.-L., Lin M.-T. (2012). Eicosapentaenoic acid and docosahexaenoic acid inhibit macrophage-induced gastric cancer cell migration by attenuating the expression of matrix metalloproteinase 10. J. Nutr. Biochem..

[146] Kalluri R., Weinberg R.A. (2009). The basics of epithelial-mesenchymal transition. J. Clin. Investig..

[147] Nishida N., Yano H., Nishida T., Kamura T., Kojiro M. (2006). Angiogenesis in cancer. Vasc. Health Risk Manag..

[148] Harris A.L. (2002). Hypoxia—A key regulatory factor in tumour growth. Nat. Rev. Cancer.

[149] Klein T., Bischoff R. (2010). Physiology and pathophysiology of matrix metalloproteases. Amino Acids.

[150] Szymczak M., Murray M., Petrovic N. (2008). Modulation of angiogenesis by omega-3 polyunsaturated fatty acids is mediated by cyclooxygenases. Blood.

[151] Suzuki I., Iigo M., Ishikawa C., Kuhara T., Asamoto M., Kunimoto T., Moore M.A., Yazawa K., Araki E., Tsuda H. (1997). Inhibitory effects of oleic and docosahexaenoic acids on lung metastasis by colon-carcinoma-26 cells are associated with reduced matrix metalloproteinase-2 and -9 activities. Int. J. Cancer.

[152] Iigo M., Nakagawa T., Ishikawa C., Iwahori Y., Asamoto M., Yazawa K., Araki E., Tsuda H. (1997). Inhibitory effects of docosahexaenoic acid on colon carcinoma 26 metastasis to the lung. Br. J. Cancer.

[153] Khojastehfard M., Dolatkhah H., Somi M.-H., Ahmad S.N.S., Estakhri R., Sharifi R., NaghiZadeh M., Rahmati-Yamchi M. (2018). The Effect of Oral Administration of PUFAs on the Matrix Metalloproteinase Expression in Gastric Adenocarcinoma Patients Undergoing Chemotherapy. Nutr. Cancer.

[154] Matsuoka T., Adair J., Lih F.B., Hsi L.C., Rubino M., E Eling T., Tomer K.B., Yashiro M., Hirakawa K., Olden K.W. (2010). Elevated dietary linoleic acid increases gastric carcinoma cell invasion and metastasis in mice. Br. J. Cancer.

[155] Angelucci A., Garofalo S., Speca S., Bovadilla A., Gravina G.L., Muzi P., Vicentini C., Bologna M. (2008). Arachidonic acid modulates the crosstalk between prostate carcinoma and bone stromal cells. Endocrine-Related Cancer.

[156] Brown M.D., Hart C.A., Gazi E., Bagley S., Clarke N.W. (2006). Promotion of prostatic metastatic migration towards human bone marrow stoma by Omega 6 and its inhibition by Omega 3 PUFAs. Br. J. Cancer.

[157] Zhang G., Panigrahy D., Mahakian L.M., Yang J., Liu J.-Y., Lee K.S.S., Wettersten H.I., Ulu A., Hu X., Tam S. (2013). Epoxy metabolites of docosahexaenoic acid (DHA) inhibit angiogenesis, tumor growth, and metastasis. Proc. Natl. Acad. Sci. USA.

[158] Bilodeau J.-F., Gevariya N., Larose J., Robitaille K., Roy J., Oger C., Galano J.-M., Bergeron A., Durand T., Fradet Y. (2021). Long chain omega-3 fatty acids and their oxidized metabolites are associated with reduced prostate tumor growth. Prostaglandins Leukot. Essent. Fat. Acids.

[159] Bai X., Shao J., Zhou S., Zhao Z., Li F., Xiang R., Zhao A.Z., Pan J. (2019). Inhibition of lung cancer growth and metastasis by DHA and its metabolite, RvD1, through miR-138-5p/FOXC1 pathway. J. Exp. Clin. Cancer Res..

[160] Khadge S., Thiele G.M., Sharp J.G., McGuire T.R., Klassen L.W., Black P.N., DiRusso C.C., Cook L., Talmadge J.E. (2018). Long-chain omega-3 polyunsaturated fatty acids decrease mammary tumor growth, multiorgan metastasis and enhance survival. Clin. Exp. Metastasis.

[161] Gonzalez-Reyes C., Marcial-Medina C., Cervantes-Anaya N., Cortes-Reynosa P., Salazar E.P. (2017). Migration and invasion induced by linoleic acid are mediated through fascin in MDA-MB-231 breast cancer cells. Mol. Cell. Biochem..

[162] Mandal C., Ghosh-Choudhury T., Dey N., Choudhury G.G., Ghosh-Choudhury N. (2012). miR-21 is targeted by omega-3 polyunsaturated fatty acid to regulate breast tumor CSF-1 expression. Carcinogenesis.

[163] Verlengia R., Gorjão R., Kanunfre C., Bordin S., Martinsdelima T., Martins E., Curi R. (2004). Comparative effects of eicosapentaenoic acid and docosahexaenoic acid on proliferation, cytokine production, and pleiotropic gene expression in Jurkat cells. J. Nutr. Biochem..

[164] Olson M.V., Liu Y.-C., Dangi B., Zimmer J.P., Salem N., Nauroth J.M. (2013). Docosahexaenoic acid reduces inflammation and joint destruction in mice with collagen-induced arthritis. Inflamm. Res..

[165] Richard C., Lewis E.D., Goruk S., Field C.J. (2016). A Dietary Supply of Docosahexaenoic Acid Early in Life Is Essential for Immune Development and the Establishment of Oral Tolerance in Female Rat Offspring. J. Nutr..

[166] Bouwens M., van de Rest O., Dellschaft N., Bromhaar M.G., De Groot L.C.P.G.M., Geleijnse J.M., Müller M., Afman L.A. (2009). Fish-oil supplementation induces antiinflammatory gene expression profiles in human blood mononuclear cells. Am. J. Clin. Nutr..

[167] Itariu B.K., Zeyda M., Hochbrugger E.E., Neuhofer A., Prager G., Schindler K., Bohdjalian A., Mascher D., Vangala S., Schranz M. (2012). Long-chain n−3 PUFAs reduce adipose tissue and systemic inflammation in severely obese nondiabetic patients: A randomized controlled trial. Am. J. Clin. Nutr..

[168] Trebble T., Arden N.K., Stroud M.A., Wootton S.A., Burdge G.C., Miles E.A., Ballinger A.B., Thompson R.L., Calder P.C. (2003). Inhibition of tumour necrosis factor-α and interleukin 6 production by mononuclear cells following dietary fish-oil supplementation in healthy men and response to antioxidant co-supplementation. Br. J. Nutr..

[169] Hudert C.A., Weylandt K.H., Lu Y., Wang J., Hong S., Dignass A., Serhan C.N., Kang J.X. (2006). Transgenic mice rich in endogenous omega-3 fatty acids are protected from colitis. Proc. Natl. Acad. Sci. USA.

[170] Kaliannan K., Li X.-Y., Wang B., Pan Q., Chen C.-Y., Hao L., Xie S., Kang J.X. (2019). Multi-omic analysis in transgenic mice implicates omega-6/omega-3 fatty acid imbalance as a risk factor for chronic disease. Commun. Biol..

[171] Xia S., Lu Y., Wang J., He C., Hong S., Serhan C.N., Kang J.X. (2006). Melanoma growth is reduced in fat-1 transgenic mice: Impact of omega-6/omega-3 essential fatty acids. Proc. Natl. Acad. Sci. USA.

[172] López-Vicario C., González-Périz A., Rius B., Morán-Salvador E., García-Alonso V., Lozano J.J., Bataller R., Cofán M., Kang J.X., Arroyo V. (2014). Molecular interplay between Δ5/Δ6 desaturases and long-chain fatty acids in the pathogenesis of non-alcoholic steatohepatitis. Gut.

[173] Warner D.R., Warner J.B., Hardesty J.E., Song Y.L., King T.N., Kang J.X., Chen C.-Y., Xie S., Yuan F., Prodhan A.I. (2019). Decreased ω-6:ω-3 PUFA ratio attenuates ethanol-induced alterations in intestinal homeostasis, microbiota, and liver injury. J. Lipid Res..

[174] Marangoni F., Agostoni C., Borghi C., Catapano A.L., Cena H., Ghiselli A., La Vecchia C., Lercker G., Manzato E., Pirillo A. (2020). Dietary linoleic acid and human health: Focus on cardiovascular and cardiometabolic effects. Atherosclerosis.

[175] Virtanen J.K., Wu J., Voutilainen S., Mursu J., Tuomainen T.-P. (2018). Serum n–6 polyunsaturated fatty acids and risk of death: The Kuopio Ischaemic Heart Disease Risk Factor Study. Am. J. Clin. Nutr..

[176] Marklund M., Wu J.H., Imamura F., Del Gobbo L.C., Fretts A., De Goede J., Shi P., Tintle N., Wennberg M., Aslibekyan S. (2019). Biomarkers of Dietary Omega-6 Fatty Acids and Incident Cardiovascular Disease and Mortality. Circulation.

[177] Wu J.H.Y., Marklund M., Imamura F., Tintle N., Korat A.V.A., de Goede J., Zhou X., Yang W.-S., Otto M.C.D.O., Kröger J. (2017). Omega-6 fatty acid biomarkers and incident type 2 diabetes: Pooled analysis of individual-level data for 39 740 adults from 20 prospective cohort studies. Lancet Diabetes Endocrinol..

[178] Liou Y.A., Innis S.M. (2009). Dietary linoleic acid has no effect on arachidonic acid, but increases n-6 eicosadienoic acid, and lowers dihomo-γ-linolenic and eicosapentaenoic acid in plasma of adult men. Prostaglandins Leukot. Essent. Fat. Acids.

[179] Moussa H., Nguile-Makao M., Robitaille K., Guertin M.-H., Allaire J., Pelletier J.-F., Moreel X., Gevariya N., Diorio C., Desmeules P. (2019). Omega-3 Fatty Acids Survey in Men under Active Surveillance for Prostate Cancer: From Intake to Prostate Tissue Level. Nutrients.

